# Land-surface processes and summer-cloud-precipitation characteristics in the Tibetan Plateau and their effects on downstream weather: a review and perspective

**DOI:** 10.1093/nsr/nwz226

**Published:** 2020-01-06

**Authors:** Yunfei Fu, Yaoming Ma, Lei Zhong, Yuanjian Yang, Xueliang Guo, Chenghai Wang, Xiaofeng Xu, Kun Yang, Xiangde Xu, Liping Liu, Guangzhou Fan, Yueqing Li, Donghai Wang

**Affiliations:** 1 School of Earth and Space Sciences, University of Science and Technology of China, Hefei 230026, China; 2 Key Laboratory of Tibetan Environment Changes and Land Surface Processes, Institute of Tibetan Plateau Research, Chinese Academy of Sciences, Beijing 100101, China; 3 CAS Center for Excellence in Tibetan Plateau Earth Sciences, Chinese Academy of Sciences, Beijing 100101, China; 4 University of Chinese Academy of Sciences, Beijing 100049, China; 5 CAS Center for Excellence in Comparative Planetology, Hefei 230026, China; 6 School of Atmospheric Physics, Nanjing University of Information Science and Technology, Nanjing 210044, China; 7 State Key Laboratory of Severe Weather, Chinese Academy of Meteorological Sciences, China Meteorological Administration, Beijing 100081, China; 8 School of Atmospheric Sciences, Lanzhou University, Lanzhou 730000, China; 9 Ministry of Education Key Laboratory for Earth System Modeling, Department of Earth System Science, Tsinghua University, Beijing 100084, China; 10 Chengdu University of Information Technology, Chengdu 610225, China; 11 Institute of Plateau Meteorology, CMA, Chengdu 610225, China; 12 School of Atmospheric Sciences, Sun Yat-sen University, Guangzhou 519082, China

**Keywords:** Tibetan Plateau, land-surface processes, cloud-precipitation characteristics, downstream effects

## Abstract

Correct understanding of the land-surface processes and cloud-precipitation processes in the Tibetan Plateau (TP) is an important prerequisite for the study and forecast of the downstream activities of weather systems and one of the key points for understanding the global atmospheric movement. In order to show the achievements that have been made, this paper reviews the progress on the observations for the atmospheric boundary layer, land-surface heat fluxes, cloud-precipitation distributions and vertical structures by using ground- and space-based multiplatform, multisensor instruments and the effect of the cloud system in the TP on the downstream weather. The results show that the form drag related to the topography, land–atmosphere momentum and scalar fluxes is an important part of the parameterization process. The sensible heat flux decreased especially in the central and northern TP caused by the decrease in wind speeds and the differences in the ground-air temperatures. Observations show that the cloud and precipitation over the TP have a strong diurnal variation. Studies also show the compressed-air column in the troposphere by the higher-altitude terrain of the TP makes particles inside clouds vary at a shorter distance in the vertical direction than those in the non-plateau area so that precipitation intensity over the TP is usually small with short duration, and the vertical structure of the convective precipitation over the TP is obviously different from that in other regions. In addition, the influence of the TP on severe weather downstream is preliminarily understood from the mechanism. It is necessary to use model simulations and observation techniques to reveal the difference between cloud precipitation in the TP and non-plateau areas in order to understand the cloud microphysical parameters over the TP and the processes of the land boundary layer affecting cloud, precipitation and weather in the downstream regions.

## Teaser

Research progresses of atmospheric boundary layer, land surface heat fluxes, cloud-precipitation distributions and vertical structures in the Tibetan Plateau (TP) by in-situ measurements, remote sensing and numerical modelling, and the effect of TP cloud system on the downstream weather are reviewed.

## INTRODUCTION

The Tibetan Plateau (TP), located in the eastern part of the subtropical Eurasia, covers an area of about a quarter of China's territory and has an average altitude of >4000 m. It has the highest altitude, largest area and most complex terrain in the world. Since the late 1970s, Chinese scientists have conducted three scientific experiments in the TP: the first Qinghai Xizang Plateau Meteorological Experiment (QXPMEX), the second Tibetan Plateau Atmospheric Experiment (TIPEX-II) and the third Tibetan Plateau Atmospheric Experiment (TIPEX-III). In 2013, the National Natural Science Foundation of China began to implement a major research project called ‘Changes in the Land-Atmosphere Coupling System on the Tibetan Plateau and Its Associated Global Climate Effects’.

In the past 40 years, some aspects of atmospheric sciences related to TP have reached consensus in phenomena and mechanisms, such as the mass transport and ozone hole caused by convections [1], an air-heating pump generated by land-surface sensible and latent heat [2,3] and a water tower due to monsoon activities [4]. Studies found that the unique and complicated boundary-layer process was the most fundamental factor producing many phenomena, including the summer prevalence of convective activities in the compressed-air column 5 km above sea level in the TP [5–8]. The thermal-forcing convective cloud in the TP usually moved eastward to downstream eastern China [9,10]. As a result, the TP is considered to be one of the important sources of convective cloud systems, significantly contributing to frequent storm rainfall and floods during summer in eastern China [11]. Chen *et al.* [12] analyzed the summer convection and precipitation over eastern China and the TP using satellite observations and the apparent heat source (Q1) and moisture sink (Q2). The positive correlation of the deep convection between eastern China and the east TP suggests that the moisture due to the evaporation of cloud water in anvil clouds detrained from the deep convection over the east TP can be transported downstream and benefit the development of convection over eastern China. In addition, the abnormal change in surface sensible heating in the TP generated the anomalous changes not only in the local circulation, but also in Asia and even in the entire northern hemisphere [13].

This article mainly summarizes and reviews recent study progress in land-surface processes and summer cloud precipitation in the TP and their impacts on downstream weather in the mainland of China, and finally gives a new perspective. The review will be organized into the following sections. After the introduction, the first part summarizes achievements in the observation and study of the atmospheric boundary layer, remote sensing of land-surface heat fluxes (LSHFs) and modeling of the precipitation and frozen–thawing process in the TP. The achievements of studies on cloud-precipitation horizontal distribution and vertical structures, cloud microphysical characteristics and precipitation in the TP are introduced in the second and third parts, respectively. Then, the achievements of the effect of the cloud system of the TP on the downstream weather are represented. Finally, the future research direction of atmospheric science on the TP is prospected.

## LAND-SURFACE PROCESSES OVER THE TP

### Retrieval of key characteristic parameters for land–atmosphere interaction

Called the roof of the world, the TP has significant impacts on atmospheric circulation and downstream weather conditions through land–atmosphere interactions [2,4]. Besides studies on the mechanical forcing of the TP in early years [14], recent research has mainly focused on the strong surface-heating status of the TP. Some consensuses about the TP heating effects (sensible heat driving an ‘air pump’) have been reached [15] and their links with the establishment and inter-annual variability of the East Asian summer monsoon (EASM) [16]. However, because of the model uncertainties and the lack of land–atmosphere interaction data, especially the shortage of high spatio-temporal land-surface heat-flux data, some debates still exist on how the thermal effects of the TP will influence the South Asian monsoon [17,18]. With the undertaking of several field experiments over the TP since 1979, such as QXPMEX, TIPEX-II, GEWEX (Global Energy and Water Cycle Experiment) Asian Monsoon Experiment on the Tibetan Plateau (GAME/Tibet), CEOP (Coordinated Enhanced Observing Period) Asia-Australia Monsoon Project (CAMP) on the Tibetan Plateau (CAMP/Tibet), Tibetan Observation and Research Platform, China-Japan Meteorological Disaster Reduction Cooperation Research Center Project (JICA/Tibet) and the ongoing project TIPEX-III, a large amount of valuable land–atmosphere interaction data sets have been retrieved and scientific understanding of land–atmosphere interactions has been achieved. However, some wide divergences still exist in the key parameters (bulk-transfer coefficients, aerodynamic roughness length (${Z_{0m}}$), thermodynamic roughness length (${Z_{0h}}$) and excess resistance to heat transfer ($\ k{B^{ - 1}}$)) [19]. The divergences will cause great uncertainties in estimation of the land-surface heat status and its impacts on the TP. Duan and Wu [20] showed a weakening trend in the TP atmospheric heat source, particularly in the spring surface sensible heat flux since the 1980s. Yang *et al*. [7] found that the annual mean sensible heat flux had a less weakened trend of 2% per decade based on a newly developed physical method. Chen *et al.* [21,22] examined the impacts of the large-scale circulation and surface-heat fluxes on the triggering and development of the cloud and precipitation over the TP, and found that the strong surface-heat flux played an important role in the heavy rainfall events over the TP. According to Wang *et al.* [19], different calculation methods may be the main reason for the opposite conclusion related to bulk-transfer coefficients. ${Z_{0m\ }}$and ${Z_{0h}}{\rm{\ }}$are two key variables in weather and climate models. For a long time, they were often thought to be the same value for simplification. Ma *et al.* [23] found that ${Z_{0m}}$ and ${Z_{0h}}$ are totally different for different land-surface types, with ${Z_{0m}}{\rm{\ }}$one order of magnitude larger than$\ {Z_{0h}}$ in the TP. The${\rm{\ }}k{B^{ - 1}}$ exhibited obvious diurnal variation over the TP. Recently, the study of Han *et al*. [24] showed that, for rugged mountainous areas in the TP, the derived effective aerodynamic roughness length ($Z_{0m}^{\mathit{eff}}$) was considerably larger than the small-scale aerodynamic roughness length. This means that the form drag exerted by the topography should be taken into account in the parameterization of land–atmosphere momentum and scalar fluxes.

### Remote sensing of LSHFs

**Figure 1.**
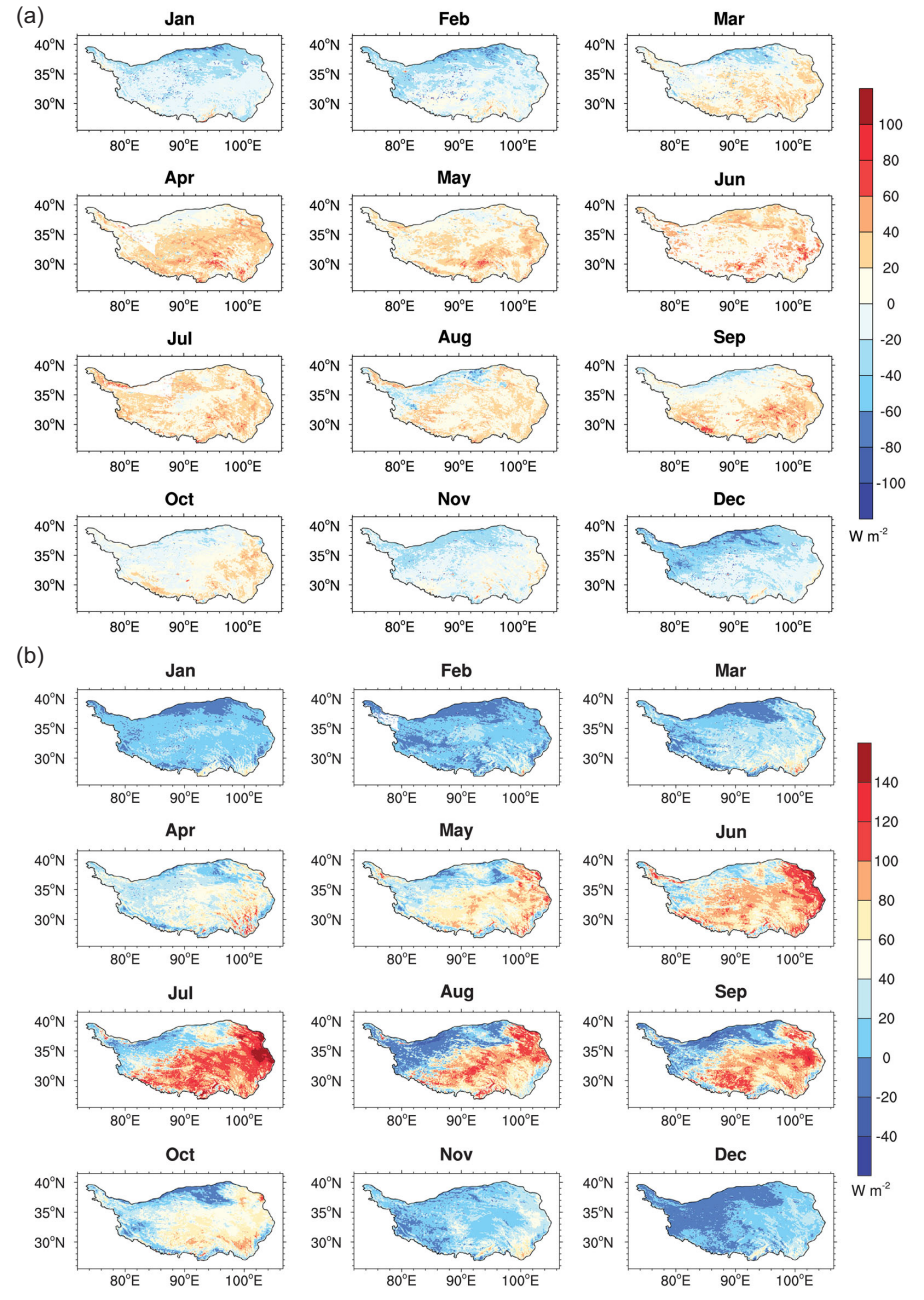
Seasonal variations in sensible heat flux (a) and laten heat flux (b) in 2008 over the TP (modified from Zhong *et al*. [28]). The spatial distribution of sensible heat flux is a little complex, while a clear spatial distribution characteristic can be identified for latent heat flux.

The LSHFs from the regional to the global scale can be derived by applying remote-sensing technology. The land-surface variables, such as land-surface temperature, albedo, vegetation index and emissivity, can be derived from satellite measurements. The LSHFs can be estimated indirectly based on the above land-surface parameters. Several approaches have been explored to derive the surface-heat fluxes at multi-spatial and temporal scales that bridge the gap between the point measurements and the regional scale [25,26]. Especially, great improvements have been made in the remote sensing of LSHFs over the TP at different spatio-temporal scales in recent years. Both polar-orbiting and geostationary satellite data have been successfully applied to derive the LSHFs over the TP. Several parameterization methodologies have been developed for different satellite sensors to derive the plateau-scale LSHFs [27]. Recently, by a combined use of geostationary and polar-orbiting satellites, hourly LSHFs for the entire TP were first estimated by Zhong *et al*. [28]. The spatio-temporal distribution of the LSHFs were consistent with the land-surface heterogeneous status and the general hydro-meteorological conditions of the TP (Fig. 1). The derived results were also found to be superior to the Global Land Data Assimilation System flux products and will help in understanding the diurnal variation of the atmospheric boundary layer [28].

**Figure 2.**
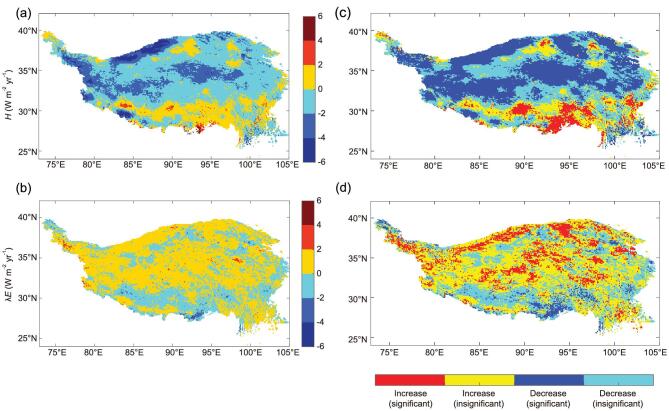
Linear trends and significance test maps of sensible heat flux *H* ((a) and (c)) and latent heat flux λ*E* ((b) and (d)) from 2001 to 2012. The trends were classified into four categories ((c) and (d)) according to statistical linear-trend analysis: a significant increase (*P* < 0.05), a significant decrease (*P* < 0.05), an insignificant increase (*P* > 0.05) and an insignificant decrease (*P* > 0.05) (modified from Han *et al*. [31]).

In the context of global warming, the rapid warming and moistening signals were identified over the TP by Zhong *et al*. [29] in recent years. Studies have found that the thermal effects of the TP had been weakening by a combination of effects of enhanced cooling through radiation loss and a decrease in the land-surface sensible heat flux [7,20]. Further analysis revealed that the decrease in the sensible heat flux over the TP will weaken monsoon circulation and postpone the seasonal reversal of the land–sea thermal contrast in East Asia [30]. A long time series of surface-energy balance components were estimated for the whole TP area from 2001 to 2012 based on satellite observations [31]. It was found that the sensible heat flux decreased overall, especially in the central and northern TP. The decreases in sensible heat flux can be explained by the decrease in wind speeds as well as differences in ground-air temperatures. The latent heat flux increased over the majority of the TP, which is attributed to increases in precipitation and vegetation greening (Fig. 2).

### Modeling of precipitation and the frozen–thawing process over the TP

The TP is one of regions with the largest precipitation bias in current climate models. It had been believed that this bias was mainly due to the fact that coarse-resolution climate modeling could not reflect the complex terrain around the plateau. Complex terrain can generate orographic drags at different scales: gravity wave drag, drag owing to low-level flow blocking and turbulence-scale drag. Theoretically, the large-scale orographic drags can be resolved by increasing the model resolution. Rahimi *et al.* [32] found that increasing the horizontal model resolution significantly decreases the modeled precipitation wet biases over the TP, by comparing the Community Earth System Model (CESM) global model at two resolutions and the regional Weather Research and Forecasting Model (WRF) simulation. However, there were still obvious precipitation errors in regional climate simulations with a resolution of 10 km [33]. The reason could be that the terrain of the Himalayan mountains is extremely complex and the turbulent drag on the air flow induced by orographic variance plays a significant role. This drag is called turbulent orographic form drag (TOFD).

**Figure 3.**
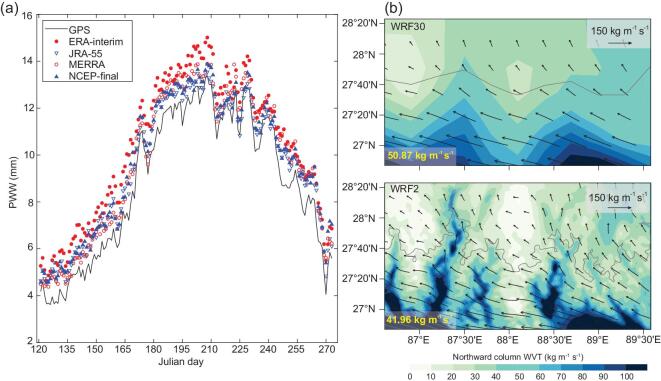
(a) Seasonal march of precipitable water between GPS-observation and four reanalyses, after elevation correction and average at nine stations during 2007–13 (after Wang *et al*. [21]); (b) spatial pattern of column water vapor flux (vector) and its meridional component (color) in simulations with 30-km-resolution (WRF30) and 2-km-resolution simulation (WRF2), with the domain average of the meridional component provided in the bottom-left corner. Gray curves represent elevation of 4000 m (modified from Lin *et al*. [35]).

Based on the observations, it is found that precipitable water vapor in current reanalysis data sets is too high and its diurnal variation is too early and too strong over the southern TP (Fig. 3a) [34]. This can be related to the coarse resolutions in the reanalysis models that are not able to resolve the complex terrain. To clarify this point, the WRF simulations with different resolutions were conducted by Lin *et al.* [35] and the simulated wind speed and water-vapor flux are shown in Fig. 3b. The simulation with a coarse resolution (30 km) yields stronger wind speed and thus brings more water vapor into the TP than the one with a fine resolution (2 km). The latter clearly shows that water vapor moves mainly through valleys but is greatly blocked by high mountains. Accordingly, the coarse-resolution simulation produces much more precipitation over the north side of the Himalayas, confirming the importance of orographic drag in this region.

**Figure 4.**
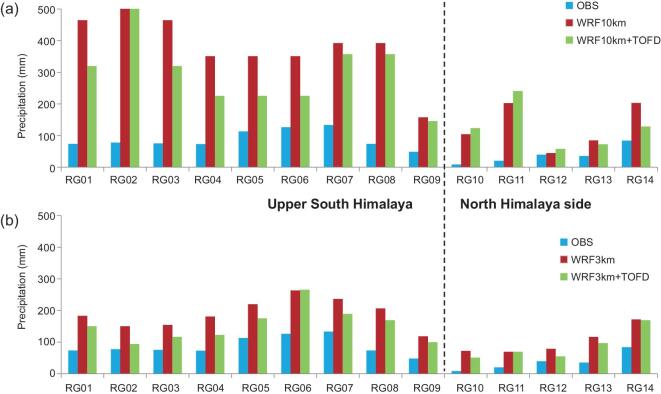
(a) Comparison of precipitation between *in situ* observation and WRF 10-km simulations w/o TOFD scheme (WRF10km, WRF10km+TOFD); (b) similar to (a) but the resolution is 3 km. The RG01–09 stations are located along the Yadong Valley of the upper South Himalayas and the RG10–14 stations are at the north side (modified from Wang *et al*. [38]).

However, it is not easy to parameterize this effect in climate models. Han *et al.* [24] clearly show that $Z_{0m}^{\mathit{eff}}$ could be in the order of 10 m, which implies that the surface layer in the atmospheric boundary layer (or the first level of the atmospheric model) must be several hundred meters if the roughness is used. However, the surface layer in the atmospheric boundary layer is usually assumed to be <100 m in current models. Alternatively, a scheme developed by Beljaars *et al.* [36] was implemented in the WRF model by Zhou *et al.* [37] to reflect the TOFD. The new scheme exerts the orographic drag on different atmospheric levels rather than changing the roughness length. Simulations with the revised WRF model show that the accuracy of the simulated wind speed and precipitation in the TP can be much improved [37]. The revised WRF

was also used to simulate precipitation in the high altitudes of the central Himalayas, where current models have very severe wet biases. Based on the observations along the Yadong Valley, we can see the wet bias is reduced greatly when the grid size varies from 10 to 3 km. Adding the TOFD scheme further reduces the precipitation bias by 50% or so at almost all stations (Fig. 4). Therefore, synergy of the TOFD scheme and high resolution can enhance the orographic drag, slow water-vapor transport and thus weaken wet biases in the terrain-complex region [38]. Yet, it is worth mentioning that the wet biases are still larger. Representation of other processes such as energy and water exchange on the complex terrain should be explored in future.

A most distinct feature over the TP is the land surface occupied by frozen ground; the land-surface processes over the TP must be affected by soil frozen–thawing (FT) that significantly affects the soil water and energy budget, and further influences the land–atmosphere interaction over and around the TP (e.g. Wang *et al.* [39]). A recent study suggested that the FT process had a water-storage effect [40]. No soil FT process could result in the drier surface (about 10% loss of soil moisture) in the after-thaw period (spring), which is caused by enhanced evaporation. The land-surface processes (i.e. land–atmosphere energy transfer) in cases of frozen soil and unfrozen soil are totally different. Variations in soil temperature and soil moisture further influence the surface diabatic heating status. Model simulation [40] shows that, without the FT process, the surface latent heat flux decreased by –1.07 ${\rm{W\ }}{{\rm{m}}^{ - 2}}$, while the surface sensible heat flux increased by 4.72 ${\rm{W\ }}{{\rm{m}}^{ - 2}}$, as the average over the whole TP.

Parameterization enhancements of soil heat and water transport in the land-surface model (LSM) have made great improvement in the simulation of terrestrial water cycles and surface-energy balances in cold regions. However, some big bias still exists in LSM simulations, especially at regions with high latitudes and complex topography [41]. To improve the simulation of the soil FT process, some improvements in the FT parameterizations in Community Land Model version 4.5 (CLM4.5) were proposed [41]. Compared to the original parameterizations in CLM, these improvements can not only reproduce the characteristics of soil moisture daily and diurnal changes, especially during the soil-thawing period, but also make the FT-process simulation much closer to the *in situ* measurements. A recent study suggests that the soil-moisture anomalies caused by the FT process can persist into the summer. These anomalies will enhance the TP’s thermal forcing to the subtropical westerlies and affect stationary Rossby wave train propagation in the middle latitudes [42].

## SUMMERTIME CLOUD OBSERVED FROM GROUND STATIONS AND SATELLITE INSTRUMENTS

Cloud amount, cloud types and their vertical structures are the main parameters for describing the macroscopic properties of clouds, while cloud diurnal variation reflects the local cloud climatology. Since the 1980s, the observations at ground stations and by satellite instruments have revealed the characteristics of the horizontal distribution and diurnal variation of cloud systems in the TP. It was found that the summer convective clouds were very small and exuberant in the TP and presented significant diurnal variation characteristics [43–45]. However, data sets from the observations at weather stations and cloud retrievals of the International Satellite Cloud Climatology Project exposed that dense cirrus, stratus, cumulus and cumulonimbus were typical types in the TP during the four seasons [46]. Convective clouds and precipitation in Nagqu usually occurred at 1100 (local standard time, hereafter the same); the development, merging and growth of local thermal-forcing convection mainly occurred at 1700–1800, and then reached the strongest intensity; since the early night, the precipitation began to weaken and continue until 0600, then convection gradually dissipated in the morning [47]. Recently, by using an hourly cloud-cover data set derived from observations of the Himawari-8 satellites, it was found that the minimum and maximum cloud fractions mainly occurred at 1000 and 1800 in the TP, respectively [48]. It will be necessary to investigate the details of the diurnal-variation amplitudes of the clouds and their regional differences in the TP, which is very important for understanding the land–atmosphere interaction process over there.

Based on the CloudSat/CALPSO data set, it was found that the frequency of clouds in the TP was 69% while the low-level cloud and middle-level cloud were 21% and 14%, respectively, mainly distributed at an altitude of 5–6 and 7–8 km. The high cloud had the smallest frequency and was distributed at an altitude of 11–12 km. There were two higher centers of cloud frequency horizontally distributed in the southeast and northwest of the TP [49,50]. Similar analysis was conducted in the east part of the TP [51]. Studies also revealed that the relative contribution rate of the single-layer cloud in the TP was higher than that in the other Asian monsoon regions [52], and large topography had a significant compression effect on cloud thickness and number of layers, resulting in the thinnest thickness of the cloud layer in the TP [53,54]. This compression effect has been found previously in precipitation profiles in the TP as viewed by the Tropical Rainfall Measuring (TRMM) precipitation radar (PR) [55]. However, observations in Nagqu ground station indicated that the frequency of low-level cloud and middle-level clouds in the TP was 48.3% and 21.9%, respectively [56]. So, the cloud frequency derived from satellite instruments needs to be further validated, and the differences in cloud frequency generated by observations from satellite instruments and ground stations need to be compared with each other. It is worth noting that the measuring manner of the CloudSat/CALPSO operated at the nadir point along the swath, which leads to a very low sampling rate. Therefore, the local cloud frequency derived by the CloudSat/CALPSO data set was not very reliable, but the cloud vertical structure given by the data set had good reliability.

Climate change of clouds in the TP was also a concern. Based on observations at 71 weather stations from the central to eastern TP in the period of 1961–2003, it was indicated that the low cloud amount exhibited a significant decreasing trend during the daytime and an increasing trend during the night-time [57]. The climate characteristics of summer convection over the TP as revealed by a geostationary satellite indicated that the convective activities advance from the southeast of the TP to the northwest with the movement of the South Asian monsoon to the TP [58]. Summer convective systems (CSs), with an average life cycle of about 36 h, contribute >60% of the total precipitation in the central-eastern TP [59].

Cloud microphysical parameters are directly related to cloud radiative forcing estimation and are key to improving cloud physical processes in weather models. During the TIPEX-III, the diurnal variations in cloud macro-parameters (cloud top, base, thickness and layer) were first obtained using integrated ground-air–space observations and the inversion algorithms on both cloud micro-parameters (cloud water/ice content) and dynamic parameters (air-ascending velocity) inside clouds were established [53,60–62]. The distributions and variations in cloud microphysical parameters inside clouds, such as the microphysical processes of supercooled water and ice-crystal growth together with phase changes, were also obtained [60–62]. It was found that deep convective clouds were mainly ice-phase and rich in small ice particles (fewer large ice crystals) in the cloud at 10 km above (below). The microphysical processes of both strong and weak deep convective clouds mainly included mixed-phase and ice-phase processes inside clouds, which provided a validation basis for the model to simulate the deep convective clouds in the TP [61]. Some cloud microphysical parameters, i.e. cloud optical thickness (COD) and cloud water path, derived from satellite retrievals showed that their variations in spatial distribution were closely associated with the increase in water-vapor transport flux divergence [63,64]. Compared with the regions around the TP, studies also showed that the range of raindrop size was broader, which led to an easy generation of precipitation by the convective cloud system in the TP [47,49]. The characteristics of cloud microphysical parameters in the TP still need to be further revealed. Therefore, an effective way to solve this issue is that the space–ground integrated observation method should be adopted combined with the simulation method using the high-resolution cloud model.

## SUMMER PRECIPITATION OBSERVED FROM GROUND STATIONS AND SATELLITE INSTRUMENTS

### Climatological changes in precipitation obtained from rain gauges

It is very difficult to obtain the spatial and temporal distribution of precipitation over the broad TP region because of the few weather stations over there. But Chinese scientists have made full use of the precipitation observations in long time series at limited ground stations to analyze the climate-change characteristics of regional precipitation [65]. Studies found that the opposite change trends existed between annual precipitation amounts and precipitation days from 1980 to 2013. The annual precipitation amounts increased with time while the precipitation days decreased [66]. The summer extreme precipitation in the middle and east of the plateau declined from southeast to northwest during 1961–2014 [67]. It was also found that the precipitation amount and frequency exhibited a bimodal pattern diurnally, occurring in the morning and evening, while the precipitation intensity had no such pattern [68]. Scientists also noted linkages between precipitation in the TP and local or non-local factors, e.g. the daily precipitation at 13 weather stations in the TP was closely linked to the strong and weak monsoon activities [69], and the spatial distribution of summer precipitation was related to the moisture transport controlled by weather systems around the TP [70].

The factors related to the precipitation intensity and its spatial and temporal distribution in the TP need to be focused on, which requires setting up more ground automatic observation systems. The method of synthetic analysis on weather processes in the TP is very important because different weather processes have their own characteristics in generating precipitations. In this regard, a study pointed out that the low-eddy-precipitation data were obtained by matching the daily low-eddy data with precipitation data at each station from 1979 to 2015 in the TP in order to explore the trend of low-eddy precipitation in the TP [71]. It will be the effective way in future study to merge multisource data for revealing the precipitation and its thermodynamic characteristics generated from activities of the different weather systems in the TP.

### Microphysical characteristics of precipitation measured by ground-based radar

It was pioneering that two digital x-band 711 radars were operated in Nagqu and Lhasa, respectively, in the first QXPMEX, which exposed the structures of convective precipitating clouds [72]. In the TIPEX-II in 1998, observations by a Doppler radar in Nagqu showed that very active convective clouds, multiple showers, thunderstorms and hail frequently occurred there. The strongest convective cloud had an echo signal of 40 dBz at a height of 14 km, and the lifetime of most convective cells was about 1 h. There was a lot of weak precipitation with prominent diurnal variation, and the maximum precipitation and atmospheric instability were in synchronous [73–76].

In the TIPEX-III in 2014, based on ground-based instruments including microwave radar, results exposed that primary cumulus and stratus often appeared at a 3-km height above the ground in Nagqu, and this height often occurred in obvious updrafts. The height of the deep convection system reached 16.5 km and there were both updraft and downdraft inside the cloud, in which there might be supercold water [77,78]. It was shown that the rising strong airflow inside the cloud carried wet snow under a $0^\circ {\rm{C}}$ layer back to the layer above again. Then, through the physical processes of sublimation, riming and clinging, this wet snow quickly grew into hail in just 10 minutes [79]. These observation data and preliminary results provide a basis for further studies on the mechanism investigation of cloud and precipitation, the improvement of the parameterization scheme for cloud and precipitation physical processes, and the retrieval algorithm development of satellite remote sensing.

Ground-based radar observation is very effective for obtaining cloud precipitation within a limited range, but it is insufficient for understanding cloud precipitation over the whole TP. With the improvement in transport, power and other facilities in the TP, it has become possible to set up a number of PRs and cloud radars there, especially in the central and western parts. In addition, ground-based radar-detection results also need to be integrated with other meteorological data, so as to reveal the corresponding thermodynamic characteristics of cloud-precipitation structures.

### Precipitation distribution in the whole TP measured by TRMM PR

The distribution of precipitation in the larger area in the TP was obtained by the measurement of the PR on board the TRMM satellite. The PR measurements exposed most TP precipitation with isolated and massive structure and stronger diurnal variation in the TP than surrounding regions [4,55,80,81], which was in line with the convective-precipitation characteristics observed by the ground stations in the past [73–78].

**Figure 5.**
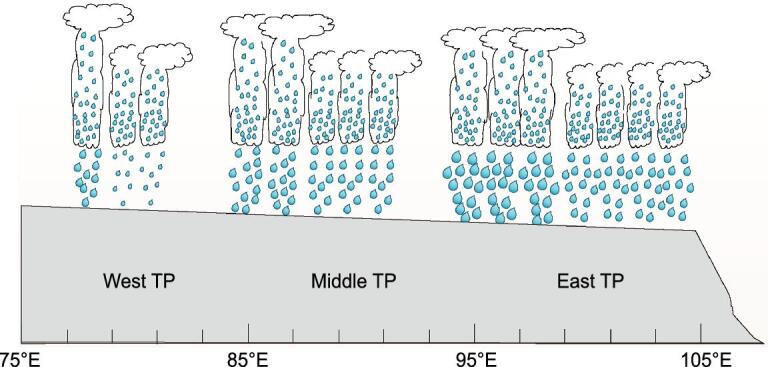
The diagrammatic sketch of the distribution for the frequency, intensity and storm-top height of precipitation from the western to eastern TP [84]. The precipitation frequency and intensity over the TP increased and strengthened from the west to the east, while the cloud-top height and echo-top height of the precipitation cloud decreased from the west to the east.

Due to the situation that the TRMM PR algorithm issued data with a large amount of stratiform precipitation in the TP, the stratiform-precipitation proportion reaching up to 80% above in most TP regions, study pointed out that the PR misjudged the strong signal reflected from the ground as one from the bright band of the stratiform so that the weak precipitation was wrongly identified as stratiform precipitation [82]. It was found that there was a relationship between the structure of the temperature/humidity profile and the depth of the precipitating cloud so that new precipitation types were defined in the TP as follows: the strong deep convective precipitation (SDCP), weak deep convective precipitation (WDCP) and shallow precipitation (SP) [83,84]. The WDCP was the dominant precipitation form (67.8%) in the TP followed by the SP (26.4%) and the SDCP (5.8%) forms, respectively. Studies also showed the time differences in frequency peak and intensity peak of both diurnal cycles for SDCP and WDCP [83,84]. Statistics indicated that the intensity and frequency of precipitation increased from the western TP towards the eastern and southeastern TP, while the contrary occurred for the storm top altitudes, as shown in Fig. 5 [83].

**Figure 6.**
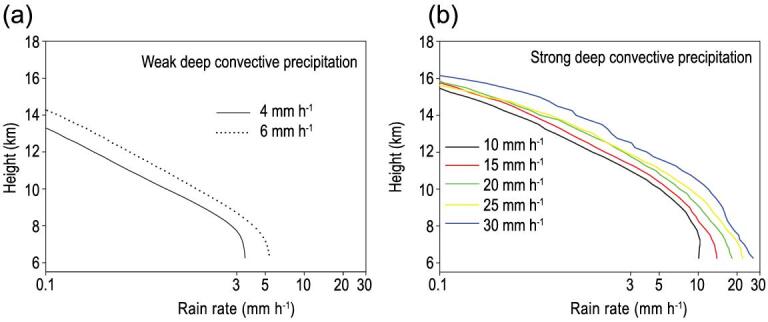
The mean profiles of the weak deep covective precipitation (WDCP) (a) and strong deep convective precipitation (SDCP) (b) in summer TP [55]. The curve in the figure represents the precipitation profiles corresponding to different ground-precipitation intensities, which indicates that they are different from those in non-plateau regions. Note: the diagram is recalculated and drawn with the data of TRMM seventh edition.

Comparative analysis showed that there were different vertical profiles between the TP and its surrounding regions. The vertical structure of the precipitation in mean profiles indicated three layers—ice or supercooled water, mixed ice and water, and droplet coalescence—for the SDCP and WDCP, as shown in Fig. 6 in which the curves represent the precipitation profiles corresponding to different ground-precipitation intensities [55], whereas, in non-plateau regions, such as East Asia or the tropical ocean, convective precipitation usually has a four-layer structure, i.e. beside the three layers, there is a layer of evaporation, in the vertical direction [85].

**Figure 7.**
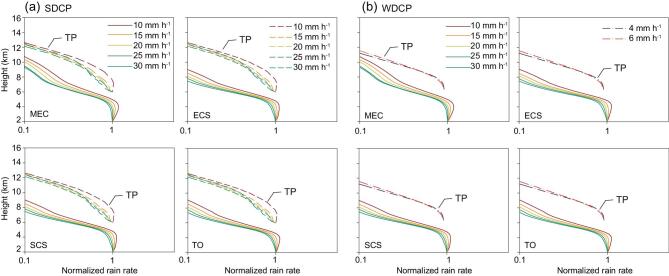
The differences between the mean profiles of the SDCP (a) / WDCP (b) in the TP and those of convection in the Mid-East China (MEC), the East China Sea (ECS), the South China Sea (SCS) and the tropical ocean (TO). The mean precipitation profiles are standardized by surface-rain rate [55]. Note: the scale of the *x*-axis is logarithmic. The diagram is recalculated and drawn with the data of TRMM seventh edition.

The differences in the precipitation profiles between the TP and the non-plateau indicated that the structure of the SDCP and WDCP in the TP was moved 6 km upwards, forced by the terrain (Fig. 7). On the other hand, the tropopause height of the TP in the summer was almost the same as that of the non-plateau area in Mid-East China (MEC) [8]. So, the thickness of the precipitation cloud was actually compressed by the terrain. The mean thicknesses of the SDCP and WDCP in the TP were about 7 and 5 km, respectively, while the mean thicknesses of the convective precipitation were up to 9 and 8 km in the MEC and in the South China Sea (SCS), respectively, while it was 7 km in both the East China Sea (ECS) and the tropical ocean (TO). Therefore, the large topography of the TP compressed the air column over it [55].

The next step of the study on the detection of precipitation in the TP should focus on two aspects: one is the comparison of the measurements of the satellite-borne PR (e.g. the Global Precipitation Measurement mission Dual-frequency Precipitation Radar) and ground-based rain radar, especially the identification of precipitation types. Due to the difference in the atmospheric environment between the TP and non-plateau areas, the precipitation structure of the TP is unique and the definition of the precipitation type in non-plateau areas is not suitable for the TP. Therefore, the characteristics of the precipitation structure of the TP should be carefully studied to give a reasonable definition of the precipitation type. The second is the evolution rule of the convective precipitation on the TP, particularly the height and thickness of the brightness band of precipitation, and the corresponding characteristics of the latent heat structure.

### Precipitation in the southern Himalayas

The south slope of the Himalayas in the summer is the region where the monsoon interacts with the steep terrain most intensively [86]. Based on the measurements of the TRMM PR, it was suggested at elevations of 1–2 km where there was a strong interaction between mesoscale CSs and steep terrain [87]. It was found that the spatio-temporal variability of precipitation ranged from the order of 1–5 km up to the continental scale [88]. Anders *et al.* [89] pointed out that there was a positive relationship between the topographic forcing factor and precipitation. Meanwhile, Shrestha *et al.* [90] suggested two remarkable rainfall peaks along the Sub-Himalayas, at about 600 m, and along the Lesser Himalayas, at about 2100 m.

Recently, Fu *et al.* [87] found that the rain frequency was the highest on the steep southern slope of the Himalayas (SSSH), while the rain intensity was highest on the foothills of the Himalayas (FHH). It was believed that the heaviest precipitation did not occur in the SSSH because most of the water vapor on the FHH has been converted into precipitation, and the residual water vapor climbed along the SSSH, so the precipitation intensity on the SSSH was small but the frequency was the highest. The result also indicated that the effects of elevation and relief have linear relationships with precipitation over the south sub-region of the SSSH, which suggested the important role of both elevation and relief on precipitation over complex plateau topography [87].

The cloud and precipitation on the SSSH need to be studied in more detail because they relate to latent heating release; for example, how do strong or weak monsoons affect the cloud and precipitation on the SSSH, and how does the advancing warm and moist monsoon affect the characteristics of the vertical structure of cloud and precipitation on the SSSH? The impact of the vertical structure of latent heat over the SSSH on atmospheric circulation is also a key factor in understanding the feedback effect of cloud precipitation.

## THE EFFECT OF THE CLOUD SYSTEM OF THE TP ON THE DOWNSTREAM WEATHER

During the boreal summer, rainstorms over the Yangtze River Basin (YRB) frequently result in flood disaster, causing substantial ramifications [91–93]. As the upstream water source of the YRB, the TP played an indispensable role in rainstorms over this region [94,95]. Xu *et al.* [96] and Wang *et al.* [94] suggested that the eastward movement of clustered convective clouds over the central part of the TP was a major cause of the rainstorms during June–July of 1998 in the YRB and found that the increased transport of atmospheric water vapor over the TP favored rainstorm formation in the middle–lower reaches of the YRB.

In phenomena, many investigations found that the eastward-moving short-wave trough and low-pressure vortex associated with the eastward-moving clouds in the TP were important factors generating the heavy rain in the eastern side of the plateau [97] and the YRB [9,98]. For example, the eastward movement of the southwest vortex along with strong convective clouds from the TP usually triggered heavy rain in the YRB [99]. Xu and Zipser [100] found that, within a range of about 1000 km eastward from the eastern part of the TP, the diurnal variation in precipitation had an obvious phase eastward-transmission phenomenon. The strong deep convection could move out of the east side of the TP (about 100°E) at 1600 local time and reached 100°E at about 2000 local time [83]. It was found that the Tibetan CS contributed to a lot of precipitation in the YRB [101]. In the TIPEX-III, observations by a C-FMCW radar site at Nagqu in the central TP showed that the vigorous convective activities were mainly distributed in this region and they frequently moved eastward [47,95]. These phenomena suggested the eastward propagation of CSs were related to the rainstorms within the YRB.

To expose the mechanism of the above phenomena, studies pointed out a good relationship between heavy rain in the YRB in 1998 and the wind field in the planetary boundary layer at the eastern edge of the TP [102]. Yasunari and Miwa [9] confirmed that the movement of convective cloud in the TP triggered a cyclonic vortex by plateau-edge cyclogenesis, and this vortex then developed into a cloud system over the downstream YRB associated with the water-vapor flow. By analyzing monthly precipitation data issued by the Global Precipitation Climatology Project and daily precipitation data observed in 438 stations in eastern China, Ge *et al*. [103] obtained that the extreme precipitation tends in the summer to be more (less) over the YRB when the TP heating is stronger (weaker) than normal. This result suggests that there is a link between the summer extreme precipitation in eastern China and the TP heating. The effects of the upper-level South Asian High (SAH) have emphasized that positive and negative vorticity anomalies in the lower and upper troposphere accompanied by an ascending motion anomaly occurred over the TP regions, respectively. The stronger heating in the TP resulted in the strengthening and eastward extension of the SAH, which led to intensity-strengthening and position-stretching westward of the western Pacific subtropical high. Therefore, there existed more extreme precipitation over the YRB due to the increase in southwestern moisture transport and moisture convergence.

The methods of the correlation analysis, WRF model simulation and Lagrangian backward tracking also confirmed the mechanism of the eastward propagation of CSs in the TP being related to the rainstorms within the YRB, such as a convective zone in the Nagqu region stimulating rainstorms in the YRB and the good relationship of the convective zone in the TP to clouds in the YRB [104,105]. The simulation also showed that the link between the deep convection in the central TP and the rainstorms downstream in the YRB was a 3D WVFV (water-vapor flux vortex) coupled structure with low-level convergence and high-level divergence. The convection would be enhanced by the eastward propagation of the WVFV structure and subsequently developed rainstorms downstream in the YRB.

In general, the above phenomena and mechanism indicate that the hourly trajectories of the simulated integral column WVFV, simulated cumulative precipitation and zonal column cloud mass are coincidentally coupled with one another, which uncovers the eastward propagation of the 3D WVFV structure originating from convection over the Nagqu region impacting on rainstorms in the downstream YRB. Studies also showed that, due to the thermodynamical forcing in the summer derived from the elevated TP topography, the frequent convective activities together with variation of cloud structures in the central TP provided significant predictive ‘strong signals’ for the occurrence and development of rainstorms over the YRB [105]. It must be pointed out that the underlying surface in the central and western parts of the TP is mostly sand and gravel, with a small heat capacity. Therefore, there exists a large temperature difference between day and night due to solar radiation. As a result, the half-day thermal forcing on convective activities and their eastward propagation should be investigated in the next step.

## SUMMARY AND FUTURE WORK

With 49 years of effort, we have been building ground observation systems to carry out experiments in the TP and analyze phenomena measured by various instruments carried by satellites. The surface sensible heat, latent heat, ground-radiation budget and so on are investigated from the local scale to the entire TP. The spatial and temporal distribution characteristics and vertical structure of cloud and precipitation in the TP are understood to a certain extent. There is also a preliminary understanding of how the plateau weather system affects the weather in the downstream regions. Because the weather systems are complicated, however, controlled by various factors, and there exists complex feedback between these systems and factors, there are still many gaps in our knowledge of atmospheric activities in the TP and their impact on the weather and climate around the TP. Studies on the land-surface and atmosphere parameters in the TP will not stop in the current study plan. It is believed that, through the unremitting efforts of several generations, we will eventually gain systematic understanding of the unique role of the TP in the global atmosphere.

So far, limitations to our recognition of the weather systems, including the land process, radiative process and cloud precipitation in the TP, mainly involve how to obtain parameters of the land surface and atmosphere, which depends on the placement of moderately dense ground instruments and the persistence of long-term observation. With abundant observation results, we can obtain detailed information on the overall atmospheric movement in the TP, and understand the thermodynamic effects on atmospheric circulation and weather processes. Although study with limited measuring stations is important, more ground observations with high temporal resolution should be put in place. Especially, some stations during the TIPEX-III only measured the atmospheric temperature, moisture and wind fields twice a day. This is certainly not sufficient for simulating the convection, cloud and precipitation processes, the diurnal variations with high-resolution models and comparisons with reanalysis data.

Satellite remote-sensing technology can make up for the shortage of ground observation to a certain extent. In order to enhance the acquisition of ground and atmospheric information in the TP, one or two geostationary meteorological satellites need to be launched to the equator facing the central part of the TP. In the meantime, multiple polar-orbiting satellites need to be launched to increase their observations with higher temporal resolution at about two hours. Polar satellites should not only carry the current passive instruments, but also carry our own PR, cloud radar and lidar to realize the 3D observations in the TP.

The authors suggest that future research will focus on the following aspects. First, the weather systems in the TP, such as the shear line, low vortex and local severe convection caused by thermodynamic circulation, should be rigorously followed, to study the variations in sensible heat, latent heat and radiation budget, including moisture, temperature and atmospheric stability, and so on, especially before and after the precipitation. That is helpful for us to understand details of the energy and water balance in the TP. The second is how to use cloud models and weather models to correctly simulate the physical processes of cloud and precipitation, for example, how cloud particles change inside the clouds and how these changes differ from those in non-plateau clouds. The third is how to get an accurate latent heat profile of cloud precipitation in the TP from the observed data in order to evaluate the latent heat structure of the model. The fourth is to understand changes in the cloud thermodynamic structure during the eastward migration of cloud clusters in the TP, and how such changes can stimulate the severe convective weather downstream. The fifth is related to investigating the climate effects of absorbing aerosols over the TP. The TP is located close to regions in South and East Asia, where the largest sources of absorbing aerosols (black carbon and dust, BCD in short) are located. The climate effects of BCD on the TP-climate system are multifold. The atmospheric BCD directly heats the atmosphere, reducing surface-incident solar radiation. On the other hand, BCD in snow on the TP could reduce the visible snow albedo by changing the surface optical properties (snow-darkening effects), which enhance the surface sensible and latent heat flux [106]. The snow-darkening effects accelerate the snowmelt, which leads to a significant positive radiative forcing and remarkably alters the regional hydrological cycle: the eastern Asian climate system [107,108]. The comprehensive effects of atmospheric BCD and deposited BCD on local radiation balance, sensible heat flux and latent heat flux is still an open scientific research issue. Furthermore, how BCD will alter the TP’s hydrological cycle through BCD–cloud-precipitation interaction needs further investigation as well.

In short, the land-surface processes of the TP and the cloud precipitation generated by these processes have very rich scientific implications, and the research results will improve our understanding of the land–atmosphere interaction over the TP and its effect on the weather downstream and even global atmospheric circulation.
